# Supplementary material for: In vitro functional analysis of gRNA sites regulating assembly of hepatitis B virus
Source: Commun Biol. 2021 Dec 16;4:1407. doi: 10.1038/s42003-021-02897-2 (PMC8677749; doi:10.1038/s42003-021-02897-2)
Supplement: Supplementary file 2 — Supplementary Information [file 42003_2021_2897_MOESM2_ESM.pdf]

Supplementary Information for:

In vitro Functional Analysis of gRNA Sites Regulating Assembly of Hepatitis B Virus.

Nikesh Patel[1*], Sam Clark[2], Eva U. Weiß[2], Carlos P. Mata[1, 4], Jen Bohon[3, 5], Erik R. Farquhar[3], Daniel P. Maskell[1], Neil A. Ranson[1], Reidun Twarock[2], & Peter G. Stockley1[*].

Supplementary Methods

Motif analysis in other packaged sequences

Three sequences were considered in this analysis: the LacZ sequence which has been shown *in vivo* not to package into HBV virions [1]; the LacZ sequence, with a 5' HBV genomic fragment containing ε which does get packaged into HBV virions[1]; and a minimal sequence shown to package into HBV virions[2]. Each of these sequences was searched for occurrences of the RGAG and GAAG Cp-binding motifs seen in the PS motifs of strain JQ707375.1. The potential secondary structures within these sequences were determined using Sfold[3], both globally across the entire sequence and locally using a sliding window of 80 nts. For both global folds and each 80 nt window 1000 sample folds were calculated. The frequencies of folds that presenting the Cp-recognition motifs in a loop was then recorded.

Sequences tested -

Epsilon (red)-LacZ sequence

GCAACTTTTTCACCTCTGCCTAATCATCTCTTGTACATGTCCCACTGTTCAAGCCTCCAAGCTGTGCCTTGGGTGGCTTTGGGGCATGGACATT
GACCCTTATAAAGAATTAATAACTACTGTGGAGTTACTCTCGTTTTTGCCTTCTGACTTCTTTCCTTCCGTCAGAGATCTCCTAGACACCGCCTC
AGCTCTGTATCGGGAAGCCTTAGAGTCTCCTGAGCATTGCTCACCTCACCACACCGCACTCAGGCAAGCCATTCTCTGCTGGGGGGAATTGAT
GACTCTAGCTACCTGTAATGGTAGCGACCGGCGCTCAGCTGGAATTCCGCCGATACTGACGGGCTCCAGGAGTCGTCGCCACCAATCCCCATA
TGGAAACCGTCGATATTCAGCCATGTGCCTTCTTCCGCGTGCAGCAGATGGCGATGGCTGGTTTCCATCAGTTGCTGTTGACTGTAGCGGCTG
ATGTTGAACTGGAAGTCGCCGCGCCACTGGTGTGGGCCATAATTCAATTCGCGCGTCCCGCAGCGCAGACCGTTTTCGCTCGGGAAGACGTA
CGGGGTATACATGTCTGACAATGGCAGATCCCAGCGGTCAAAACAGGCGGCAGTAAGGCGGTCGGGATAGTTTTCTTGCGGCCCTAATCCGA
GCCAGTTTACCCGCTCTGCTACCTGCGCCAGCTGGCAGTTCAGGCCAATCCGCGCCGGATGCGGTGTATCGCTCGCCACTTCAACATCAACGG
TAATCGCCATTTGACCACTACCATCAATCCGGTAGGTTTTCCGGCTGATAAATAAGGTTTTCCCCTGATGCTGCCACGCGTGAGCGGTCGTAAT
CAGCACCGCATCAGCAAGTGTATCTGCCGTGCACTGCAACAACGCTGCTTCGGCCTGGTAATGGCCCGCCGCCTTCCAGCGTTCGACCCAGG
CGTTAGGGTCAATGCGGGTCGCTTCACTTACGCCAATGTCGTTATCCAGCGGTGCACGGGTGAACTGATCGCGCAGCGGCGTCAGCAGTTGT
TTTTTATCGCCAATCCACATCTGTGAAAGAAAGCCTGACTGGCGGTTAAATTGCCAACGCTTATTACCCAGCTCGATGCAAAAATCCATTTCGCT
GGTGGTCAGATGCGGGATGGCGTGGGACGCGGCGGGGAGCGTCACACTGAGGTTTTCCGCCAGACGCCACTGCTGCCAGGCGCTGATGTGC
CCGGCTTCTGACCATGCGGTCGCGTTCGGTTGCACTACGCGTACTGTGAGCCAGAGTTGCCCGGCGCTCTCCGGCTGCGGTAGTTCAGGCAG
TTCAATCAACTGTTTACCTTGTGGAGCGACATCCAGAGGCACTTCACCGCTTGCCAGCGGCTTACCATCCAGCGCCACCATCCAGTGCAGGAG
CTCGTTATCGCTATGACGGAACAGGTATTCGCTGGTCACTTCGATGGTTTGCCCGGATAAACGGAACTGGAAAAACTGCTGCTGGTGTTTTGC
TTCCGTCAGCGCTGGATGCGGCGTGCGGTCGGCAAAGACCAGACCGTTCATACAGAACTGGCGATCGTTCGGCGTATCGCCAAAATCACCGC
CGTAAGCCGACCACGGGTTGCCGTTTTCATCATATTTAATCAGCGACTGATCCACCCAGTCCCAGACGAAGCCGCCCTGTAAACGGGGATACT
GACGAAACGCCTGCCAGTATTTAGCGAAACCGCCAAGACTGTTACCCATCGCGTGGGCGTATTCGCAAAGGATCAGCGGGCGCGTCTCTCCA
GGTAGCGAAAGCCATTTTTTGATGGACCATTTCGGCACAGCCGGGAAGGGCTGGTCTTCATCCACGCGCGCGTACATCGGGCAAATAATATCG
GTGGCCGTGGTGTCGGCTCCGCCGCCTTCATACTGCACCGGGCGGGAAGGATCGACAGATTTGATCCAGCGATACAGCGCGTCGTGATTAGC
GCCGTGGCCTGATTCATTCCCCAGCGACCAGATGATCACACTCGGGTGATTACGATCGCGCTGCACCATTCGCGTTACGCGTTCGCTCATCGC
CGGTAGCCAGCGCGGATCATCGGTCAGACGATTCATTGGCACCATGCCGTGGGTTTCAATATTGGCTTCATCCACCACATACAGGCCGTAGCG
GTCGCACAGCGTGTACCACAGCGGATGGTTCGGATAATGCGAACAGCGCACGGCGTTAAAGTTGTTCTGCTTCATCAGCAGGATATCCTGCAC
CATCGTCTGCTCATCCATGACCTGACCATGCAGAGGATGATGCTCGTGACGGTTAACGCCTCGAATCAGCAACGGCTTGCCGTTCAGCAGCAG
CAGACCATTTTCAATCCGCACCTCGCGGAAACCGACATCGCAGGCTTCTGCTTCAATCAGCGTGCCGTCGGCGGTGTGCAGTTCAACCACCGC
ACGATAGAGATTCGGGATTTCGGCGCTCCACAGTTTCGGGTTTTCGACGTTCAGACGTAGTGTGACGCGATCGGCATAACCACCACGCTCATC
GATAATTTCACCGCCGAAAGGCGCGGTGCCGCTGGCGACCTGCGTTTCACCCTGCCATAAAGAAACTGTTACCCGTAGGTAGTCACGCAACTC
GCCGCACATCTGAACTTCAGCCTCCAGTACAGCGCGGCTGAAATCATCATTAAAGCGAGTGGCAACATGGAAATCGCTGATTTGTGTAGTCGG
TTTATGCAGCAACGAGACGTCACGGAAAATGCCGCTCATCCGCCACATATCCTGATCTTCCAGATAACTGCCGTCACTCCAGCGCAGCACCATC
ACCGCGAGGCGGTTTTCTCCGGCGCGTAAAAATGCGCTCAGGTCAAATTCAGACGGCAAACGACTGTCCTGGCCGTAACCGACCCAGCGCCC
GTTGCACCACAGATGAAACGCCGAGTTAACGCCATCAAAAATAATTCGCGTCTGGCCTTCCTGTAGCCAGCTTTCATCAACATTAAATGTGAGC
GAGTAACAACCCGTCGGATTCTCCGTGGGAACAAACGGCGGATTGACCGTAATGGGATAGGTCACGTTGGTGTAGATGGGCGCATCGTAACC

GTGCATCTGCCAGTTTGAGGGGACGACGACAGTATCGGCCTCAGGAAGATCGCACTCCAGCCAGCTTTCCGGCACCGCTTCTGGTGCCGGAA
ACCAGGCAAAGCGCCATTCGCCATTCAGGCTGCGCAACTGTTGGGAAGGGCGATCGGTGCGGGCCTCTTCGCTATTACGCCAGCTGGCGAAA
GGGGGATGTGCTGCAAGGCGATTAAGTTGGGTAACGCCAGGGTTTTCCCAGTCACGACGTTGTAAAACGACGGCCAGTGAATCCGTAATCAT
GGTCAT

LacZ sequence

TAATGGTAGCGACCGGCGCTCAGCTGGAATTCCGCCGATACTGACGGGCTCCAGGAGTCGTCGCCACCAATCCCCATATGGAAACCGTCGATA
TTCAGCCATGTGCCTTCTTCCGCGTGCAGCAGATGGCGATGGCTGGTTTCCATCAGTTGCTGTTGACTGTAGCGGCTGATGTTGAACTGGAAG
TCGCCGCGCCACTGGTGTGGGCCATAATTCAATTCGCGCGTCCCGCAGCGCAGACCGTTTTCGCTCGGGAAGACGTACGGGGTATACATGTCT
GACAATGGCAGATCCCAGCGGTCAAAACAGGCGGCAGTAAGGCGGTCGGGATAGTTTTCTTGCGGCCCTAATCCGAGCCAGTTTACCCGCTC
TGCTACCTGCGCCAGCTGGCAGTTCAGGCCAATCCGCGCCGGATGCGGTGTATCGCTCGCCACTTCAACATCAACGGTAATCGCCATTTGACC
ACTACCATCAATCCGGTAGGTTTTCCGGCTGATAAATAAGGTTTTCCCCTGATGCTGCCACGCGTGAGCGGTCGTAATCAGCACCGCATCAGCA
AGTGTATCTGCCGTGCACTGCAACAACGCTGCTTCGGCCTGGTAATGGCCCGCCGCCTTCCAGCGTTCGACCCAGGCGTTAGGGTCAATGCG
GGTCGCTTCACTTACGCCAATGTCGTTATCCAGCGGTGCACGGGTGAACTGATCGCGCAGCGGCGTCAGCAGTTGTTTTTTATCGCCAATCCA
CATCTGTGAAAGAAAGCCTGACTGGCGGTTAAATTGCCAACGCTTATTACCCAGCTCGATGCAAAAATCCATTTCGCTGGTGGTCAGATGCGG
GATGGCGTGGGACGCGGCGGGGAGCGTCACACTGAGGTTTTCCGCCAGACGCCACTGCTGCCAGGCGCTGATGTGCCCGGCTTCTGACCAT
GCGGTCGCGTTCGGTTGCACTACGCGTACTGTGAGCCAGAGTTGCCCGGCGCTCTCCGGCTGCGGTAGTTCAGGCAGTTCAATCAACTGTTT
ACCTTGTGGAGCGACATCCAGAGGCACTTCACCGCTTGCCAGCGGCTTACCATCCAGCGCCACCATCCAGTGCAGGAGCTCGTTATCGCTATG
ACGGAACAGGTATTCGCTGGTCACTTCGATGGTTTGCCCGGATAAACGGAACTGGAAAAACTGCTGCTGGTGTTTTGCTTCCGTCAGCGCTGG
ATGCGGCGTGCGGTCGGCAAAGACCAGACCGTTCATACAGAACTGGCGATCGTTCGGCGTATCGCCAAAATCACCGCCGTAAGCCGACCACG
GGTTGCCGTTTTCATCATATTTAATCAGCGACTGATCCACCCAGTCCCAGACGAAGCCGCCCTGTAAACGGGGATACTGACGAAACGCCTGCC
AGTATTTAGCGAAACCGCCAAGACTGTTACCCATCGCGTGGGCGTATTCGCAAAGGATCAGCGGGCGCGTCTCTCCAGGTAGCGAAAGCCATT
TTTTGATGGACCATTTCGGCACAGCCGGGAAGGGCTGGTCTTCATCCACGCGCGCGTACATCGGGCAAATAATATCGGTGGCCGTGGTGTCG
GCTCCGCCGCCTTCATACTGCACCGGGCGGGAAGGATCGACAGATTTGATCCAGCGATACAGCGCGTCGTGATTAGCGCCGTGGCCTGATTCA
TTCCCCAGCGACCAGATGATCACACTCGGGTGATTACGATCGCGCTGCACCATTCGCGTTACGCGTTCGCTCATCGCCGGTAGCCAGCGCGGA
TCATCGGTCAGACGATTCATTGGCACCATGCCGTGGGTTTCAATATTGGCTTCATCCACCACATACAGGCCGTAGCGGTCGCACAGCGTGTACC
ACAGCGGATGGTTCGGATAATGCGAACAGCGCACGGCGTTAAAGTTGTTCTGCTTCATCAGCAGGATATCCTGCACCATCGTCTGCTCATCCAT
GACCTGACCATGCAGAGGATGATGCTCGTGACGGTTAACGCCTCGAATCAGCAACGGCTTGCCGTTCAGCAGCAGCAGACCATTTTCAATCCG
CACCTCGCGGAAACCGACATCGCAGGCTTCTGCTTCAATCAGCGTGCCGTCGGCGGTGTGCAGTTCAACCACCGCACGATAGAGATTCGGGA
TTTCGGCGCTCCACAGTTTCGGGTTTTCGACGTTCAGACGTAGTGTGACGCGATCGGCATAACCACCACGCTCATCGATAATTTCACCGCCGAA
AGGCGCGGTGCCGCTGGCGACCTGCGTTTCACCCTGCCATAAAGAAACTGTTACCCGTAGGTAGTCACGCAACTCGCCGCACATCTGAACTTC
AGCCTCCAGTACAGCGCGGCTGAAATCATCATTAAAGCGAGTGGCAACATGGAAATCGCTGATTTGTGTAGTCGGTTTATGCAGCAACGAGAC
GTCACGGAAAATGCCGCTCATCCGCCACATATCCTGATCTTCCAGATAACTGCCGTCACTCCAGCGCAGCACCATCACCGCGAGGCGGTTTTC
TCCGGCGCGTAAAAATGCGCTCAGGTCAAATTCAGACGGCAAACGACTGTCCTGGCCGTAACCGACCCAGCGCCCGTTGCACCACAGATGAA
ACGCCGAGTTAACGCCATCAAAAATAATTCGCGTCTGGCCTTCCTGTAGCCAGCTTTCATCAACATTAAATGTGAGCGAGTAACAACCCGTCGG
ATTCTCCGTGGGAACAAACGGCGGATTGACCGTAATGGGATAGGTCACGTTGGTGTAGATGGGCGCATCGTAACCGTGCATCTGCCAGTTTGA
GGGGACGACGACAGTATCGGCCTCAGGAAGATCGCACTCCAGCCAGCTTTCCGGCACCGCTTCTGGTGCCGGAAACCAGGCAAAGCGCCATT
CGCCATTCAGGCTGCGCAACTGTTGGGAAGGGCGATCGGTGCGGGCCTCTTCGCTATTACGCCAGCTGGCGAAAGGGGGATGTGCTGCAAG
GCGATTAAGTTGGGTAACGCCAGGGTTTTCCCAGTCACGACGTTGTAAAACGACGGCCAGTGAATCCGTAATCATGGTCAT

Minimal Junker sequence[2]

CCGCTGTCTCCACCTTTGAGAAACACTCATCCTCAGGCCATGCAGTAATTCCACAACCTTCCACCAAACTCTGCAAGATCCCAGAGTGAGAGG
CCTGTATTTCCCTGCTGGTGGCTCCAGTTCAGGAACAGTAA

Supplementary Figures

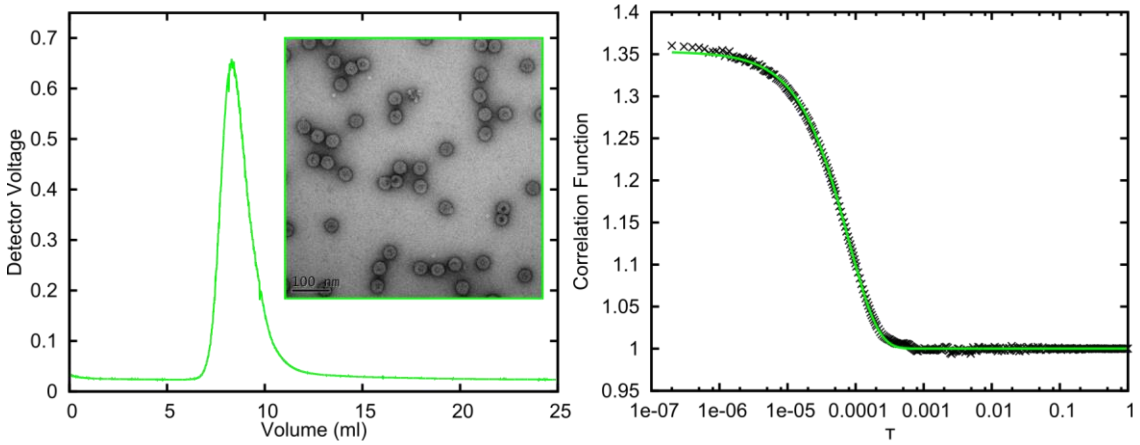

Supplementary Figure 1: Light-scattering, gel filtration signals from HBV NCPs formed in E. coli.

LS and Rh data from SEC-MALLS analysis of HBV NCPs formed up on Cp expression in *E. coli* (Rh = 19.4 nm). Data found in Sup Data S1.

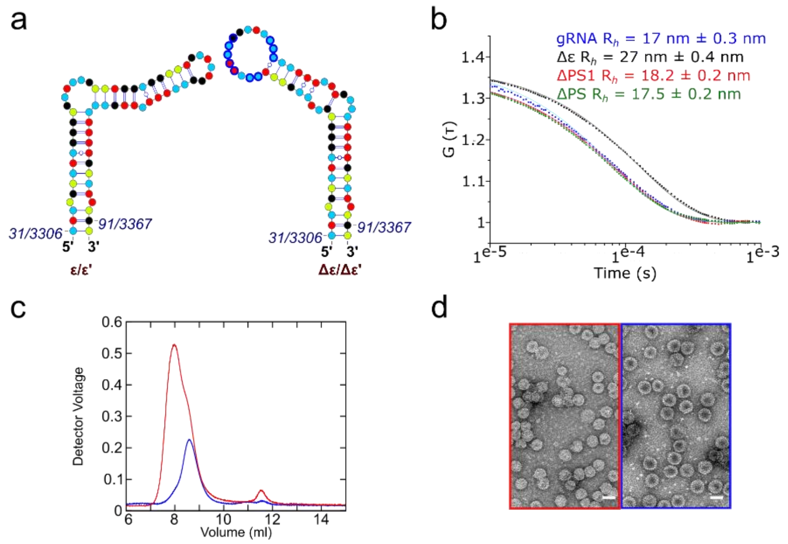

Supplementary Figure 2: Reassembly of NCPs with Δε variant pgRNA.

S-fold secondary structures of the regions surrounding: (a) the wild-type JQ707375.1 ε/ ε' *(left)* and the Δε variant *(right)*. RNA nucleotides are shown as in Fig 2. (b) Autocorrelation curves for the JQ707375.1 transcript (*blue*), and the ΔPS (*green*), ΔPS1 (*red*) and Δε (*black*) variants measured by SEC-MALLs. The derived R_h values for these RNAs: gRNA transcript, ΔPS,

ΔPS1 and Δε, are 17 ± 0.3, 17.5 ± 0.2, 18.2 ± 0.2 and 27± 0.4 nm, respectively. (c) Result of an *in vitro* reassembly of the Δε variant, as described for the gRNA transcript (Fig. 3A). (d) *Left / Right:* Colour-coded nsEMs of products from (c). Scale bars = 50 nm. Data found in Sup Data S2.

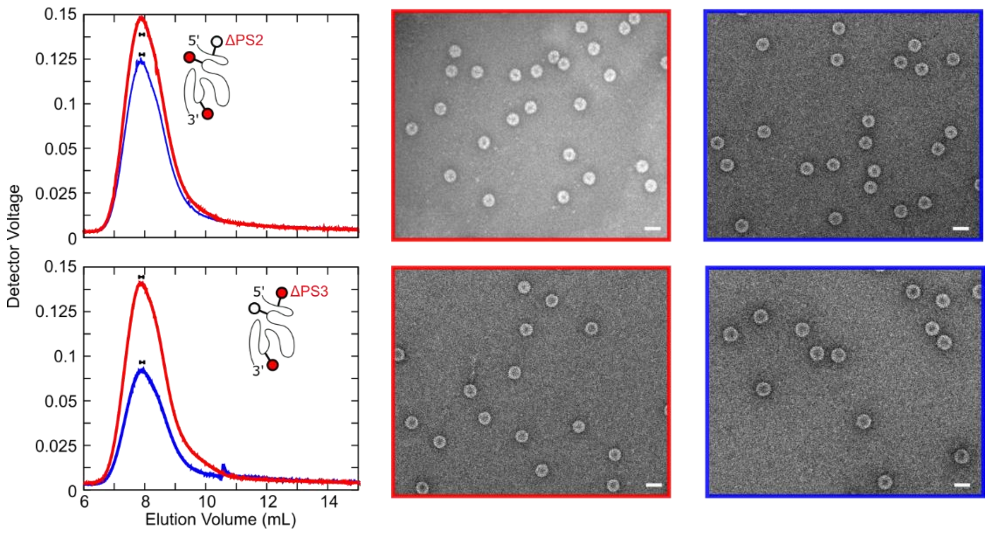

Supplementary Figure 3: Reassembly of NCPs with pgRNAs containing variants of PS2 or PS3.

NCP reassemblies containing 1 nM pgRNA with ΔPS2 (top) or ΔPS3 (bottom) and HBV Cp dimer titrations, see Fig 2a for details. LS traces before (red) or after (blue) 1 μM RNase A treatment. *Middle / Right panels:* nsEMs of re-assembly products, colour-coded as in LS traces**.** Scale bars = 50 nm. Data found in Sup Data S3.

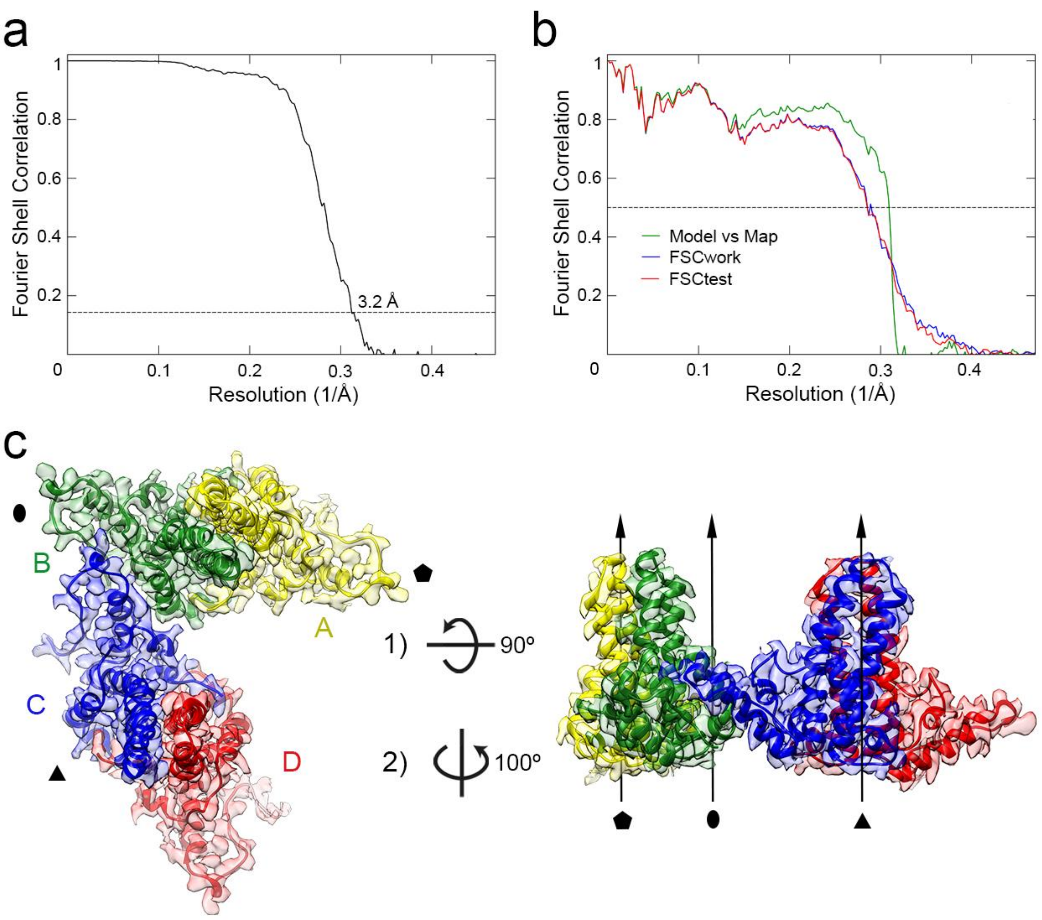

Supplementary Figure 4: Resolution and model validation of HBV *T*=4 NCP structure.

(a) Fourier Shell Correlation (FSC) resolution curve for the icosahedrally-averaged 3D reconstruction of HBV *T*=4 NCP. Resolution based on the gold standard 0.143 criterion is 3.2 Å. (b) Cross-validation against overfitting of the model. The FSC curve for the final atomic model refined against the post-processed map (green curve, Model vs Map), and FSC curves for the randomly shifted and refined atomic model against the half map used in the refinement (blue curve, FSCwork) and against the half map not used in the refinement (red curve, FSCtest). (c) Atomic model of the asymmetric unit of HBV *T*=4 NCP shown as ribbon diagrams (top view, left; side view, right) colour-coded as in Fig 5, fitted into the 3.2 Å resolution cryo-EM density map shown as colour-coded semi-transparent surface. Symbols and arrows indicate icosahedral symmetry axes.

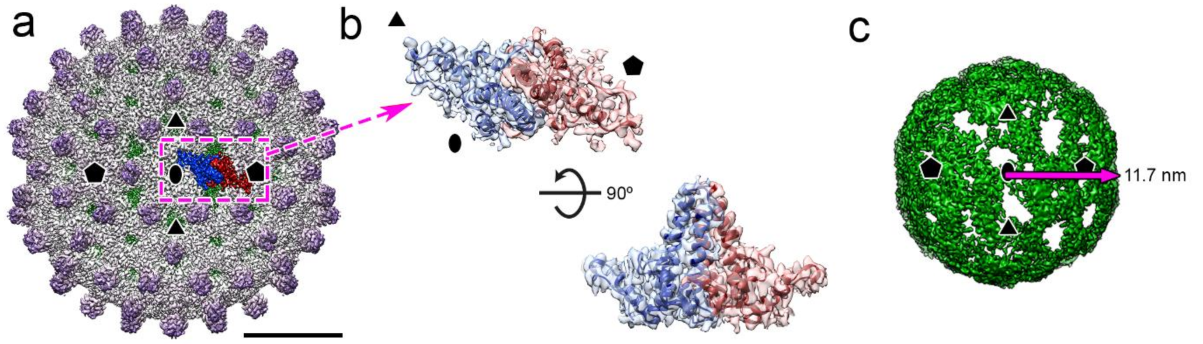

Supplementary Figure 5: Cryo-EM reconstruction of the *T*=4 NCP formed with the gRNA transcript.

(a) Front-half of the icosahedrally-averaged cryo-EM density map of the reassembled HBV *T*=4 NCP containing the wild-type JQ707375.1 RNA transcript at 3.2 Å resolution (bar = 100 Å). The dashed box surrounds a Cp dimer, subunits highlighted in blue and red, shown in (b) with top and side views of the dimer (PDB: 7ABL) fitted into the map segmented from (a). (c) A randomly selected class (Class 3) obtained after symmetry expansion and focused classification of the internal density of the structure in (a) low-pass filtered to 5 Å resolution. The arrow indicates the radius of this feature. Maps are radially colour-coded (green-white-purple), shown at 2σ, and viewed along a two-fold axis.

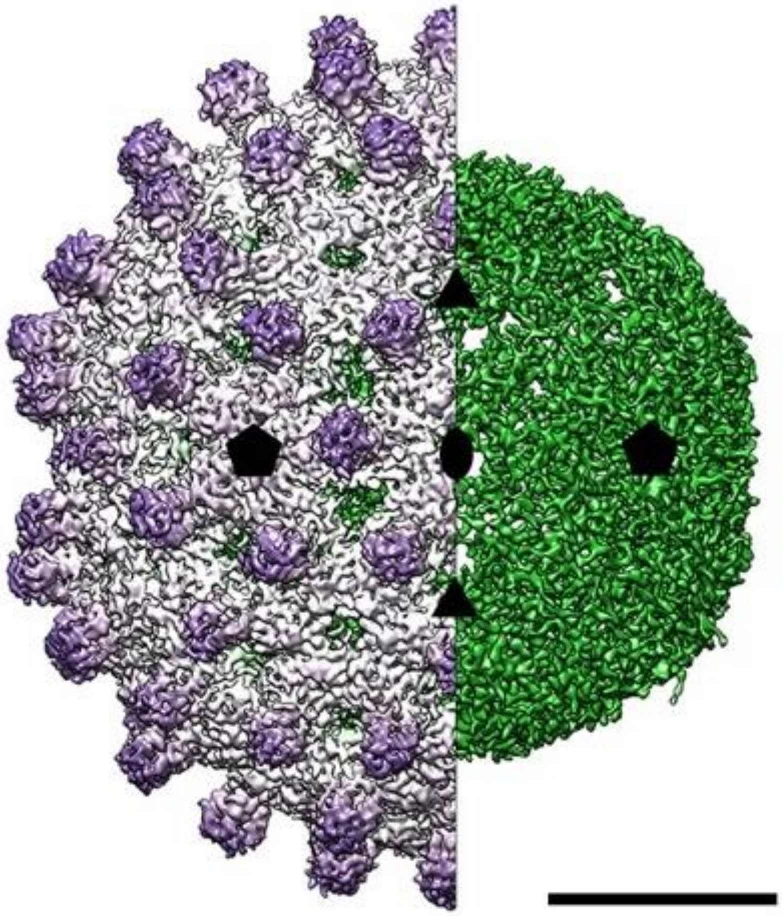

Supplementary Figure 6: Asymmetric reconstruction of the *T*=4 NCP.

Asymmetric cryo-EM density map of HBV *T*=4 NCP reconstructed at 4.2 Å resolution without imposition of icosahedral symmetry (right half, internal density in Sup Fig 5 low-pass filtered to 5 Å resolution). Bar = 100 Å.

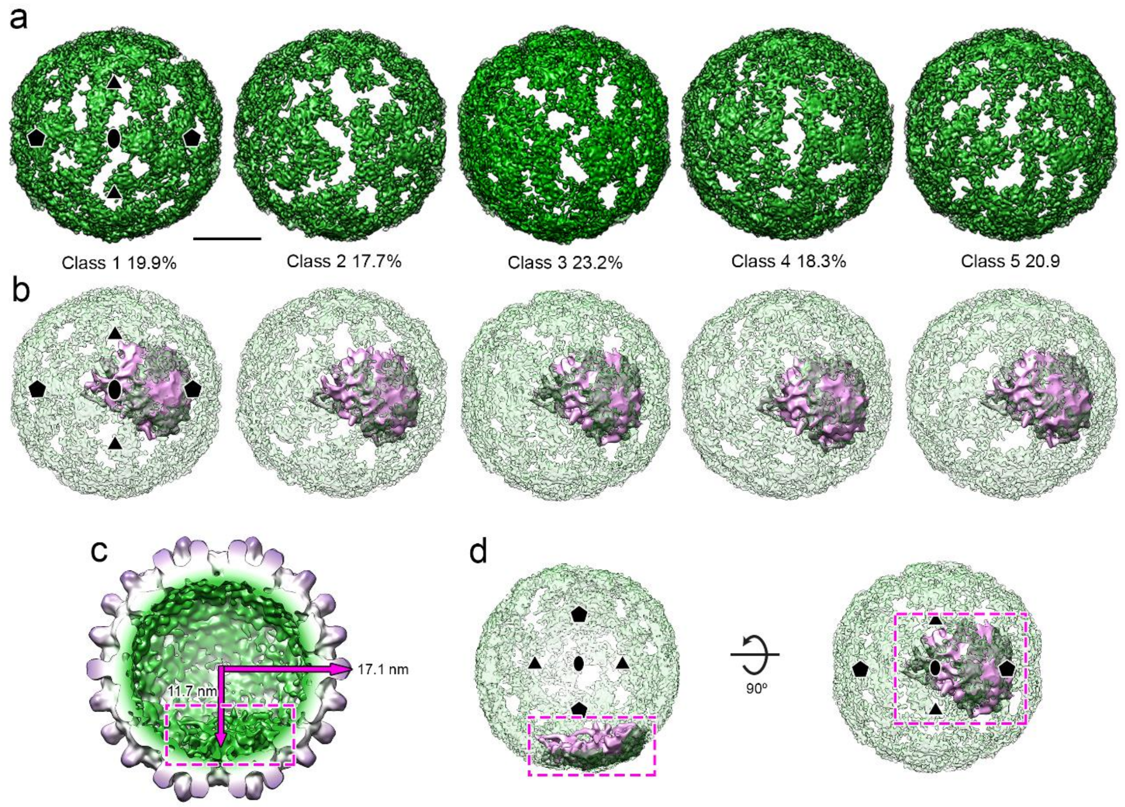

Supplementary Figure 7: Evidence that the internal density may include the RNA PSs. (a) Symmetry expansion and focused classification of the internal density (right half, low-pass filtered to 5 Å resolution) of the structure in Figure 4. All particles fit in one of five, equally-populated similar classes, shown below. Bar = 100 Å. Symbols indicate icosahedral symmetry axes. (b) Superposition of the density corresponding to the asymmetric feature seen in NC_003977.1 NCPs assembled around an oligonucleotide encompassing the PS1 from that strain (pink) into the internal density (transparent green) from classes 1 to 5[4]. (c) Back-half of the asymmetric cryo-EM density map of PS1 containing HBV VLP at 11.4 Å resolution (EMD-3714). Dashed box indicates the density corresponding to the feature observed in the NCP formed around PS1 (b). Arrows indicate the radii of the NCP and the internal shell. (d) Detail of the superposition of density corresponding to the PS1 NCP (pink) into internal shell (transparent green) of density from Class 3. Maps are radially colour-coded as in Fig 4, shown at 2σ and viewed along a two-fold axis.

**Motif position**	**Motif sequence**	**Motif Region**	**Loop expression frequency**	
			**Global folding**	**Local folding**
124	GGAG	Epsilon	1	15
167	AGAG	Epsilon	0	76
203	GAAG	Epsilon	76	1732
211	AGAG	Epsilon	3	104
351	GGAG	LacZ	0	0
480	GAAG	LacZ	0	10
552	GAAG	LacZ	0	289
1154	GGAG	LacZ	0	48
1262	AGAG	LacZ	4	6
1324	GGAG	LacZ	0	14
1336	AGAG	LacZ	0	32
1391	GGAG	LacZ	1	0
1647	GAAG	LacZ	2	100
1809	GAAG	LacZ	59	2958
1903	GAAG	LacZ	0	10
2260	AGAG	LacZ	0	5
2420	AGAG	LacZ	3	496
3110	GAAG	LacZ	0	11
3202	GAAG	LacZ	0	1556

Supplementary Table 1: RGAG and GAAG motifs found in Epsilon+LacZ. Left to right: position of motif in the sequence; motif sequence; region in which the motif is located; number of times this motif is expressed in a loop when the entire sequence is folded with Sfold; number of times this motif is expressed in a loop when the sequence is folded locally with Sfold using a sliding window of 80 nts.

Motif position	Motif sequence	Loop expression frequency	
		Global folding	Local folding
54	GGAG	0	2
183	GAAG	0	10
255	GAAG	0	245
857	GGAG	0	86
965	AGAG	0	9
1027	GGAG	0	3
1039	AGAG	0	31
1094	GGAG	0	2
1350	GAAG	1	57
1512	GAAG	63	2404
1606	GAAG	0	9
1963	AGAG	0	4
2123	AGAG	1	482
2813	GAAG	0	5
2905	GAAG	0	1147

Supplementary Table 2: RGAG and GAAG motifs found in LacZ. Left to right: position of motif in the sequence, motif sequence, number of times this motif is expressed in a loop when the entire sequence is folded with Sfold, number of times this motif is expressed in a loop when the sequence is folded locally with Sfold using a sliding window of 80 nts.

Motif position	Motif sequence	Loop expression frequency	
		Global folding	Local folding
83	AGAG	0	79
89	AGAG	0	124

Supplementary Table 3: RGAG motifs found in Junker minimal sequence. Left to right: position of motif in the sequence, motif sequence, number of times this motif is expressed in a loop when the entire sequence is folded with Sfold, number of times this motif is expressed in a loop when the sequence is folded locally with Sfold using a sliding window of 80 nts.

Supplementary References

1. Pollack, J. R. & Ganem, D. An RNA stem-loop structure directs hepatitis B virus genomic RNA encapsidation. *J. Virol.* (1993) doi:10.1128/jvi.67.6.3254-3263.1993.
2. Junker-Niepmann, M., Bartenschlager, R. & Schaller, H. A short cis-acting sequence is required for hepatitis B virus pregenome encapsidation and sufficient for packaging of foreign RNA. *EMBO J.* (1990) doi:10.1002/j.1460-2075.1990.tb07540.x.
3. Ding, Y., Chan, C. Y. & Lawrence, C. E. Sfold web server for statistical folding and rational design of nucleic acids. *Nucleic Acids Res.* (2004) doi:10.1093/nar/gkh449.
4. Patel, N. *et al.* HBV RNA pre-genome encodes specific motifs that mediate interactions with the viral core protein that promote nucleocapsid assembly. *Nat. Microbiol.* **2**, 17098 (2017).
