# Supplementary material for: In vitro functional analysis of gRNA sites regulating assembly of hepatitis B virus
Source: Commun Biol. 2021 Dec 16;4:1407. doi: 10.1038/s42003-021-02897-2 (PMC8677749; doi:10.1038/s42003-021-02897-2)
Supplement: Supplementary file 3 — Description of Additional Supplementary Files [file 42003_2021_2897_MOESM3_ESM.docx]

**Description of Additional Supplementary Files**

**File name:** Supplementary Data 1

**Description:** Light scattering data for NCP reassemblies around gRNA and ΔPS/PS1.

**File name:** Supplementary Data 2

**Description:** Normalised reactivity and ΔReactivity values plotted in Fig 5b & d.

**File name:** Supplementary Data S1

**Description:** Light scattering data and autocorrelation curve of NCPs expressed in *E. coli*.

**File name:** Supplementary Data S2

**Description:** Data for autocorrelation curves of gRNA, Δε, ΔPS and ΔPS1; and light scattering as a result of NCP reassemblies around Δε.

**File name:** Supplementary Data S3

**Description:** Light scattering data for NCP reassemblies around ΔPS2/3.
