# Supplementary material for: In vitro functional analysis of gRNA sites regulating assembly of hepatitis B virus
Source: Commun Biol. 2021 Dec 16;4:1407. doi: 10.1038/s42003-021-02897-2 (PMC8677749; doi:10.1038/s42003-021-02897-2)
Supplement: Supplementary file 9 — Reporting Summary [file 42003_2021_2897_MOESM9_ESM.pdf]

nature portfolio

Corresponding author(s): Nikesh Patel
Peter Stockley

Last updated by author(s): Oct 24, 2021

Reporting Summary

Nature Portfolio wishes to improve the reproducibility of the work that we publish. This form provides structure for consistency and transparency in reporting. For further information on Nature Portfolio policies, see our Editorial Policies and the Editorial Policy Checklist.

Statistics

For all statistical analyses, confirm that the following items are present in the figure legend, table legend, main text, or Methods section.

n/a	Confirmed	
☐	☒	The exact sample size (n) for each experimental group/condition, given as a discrete number and unit of measurement
☐	☒	A statement on whether measurements were taken from distinct samples or whether the same sample was measured repeatedly
☒	☐	The statistical test(s) used AND whether they are one- or two-sided<br>*Only common tests should be described solely by name; describe more complex techniques in the Methods section.*
☒	☐	A description of all covariates tested
☐	☒	A description of any assumptions or corrections, such as tests of normality and adjustment for multiple comparisons
☐	☒	A full description of the statistical parameters including central tendency (e.g. means) or other basic estimates (e.g. regression coefficient) AND variation (e.g. standard deviation) or associated estimates of uncertainty (e.g. confidence intervals)
☒	☐	For null hypothesis testing, the test statistic (e.g. F, t, r) with confidence intervals, effect sizes, degrees of freedom and P value noted<br>*Give P values as exact values whenever suitable.*
☒	☐	For Bayesian analysis, information on the choice of priors and Markov chain Monte Carlo settings
☒	☐	For hierarchical and complex designs, identification of the appropriate level for tests and full reporting of outcomes
☒	☐	Estimates of effect sizes (e.g. Cohen's d, Pearson's r), indicating how they were calculated

Our web collection on statistics for biologists contains articles on many of the points above.

Software and code

Policy information about availability of computer code

Data collection	Cryo EM data acquisition was performed with EPU Automated Data Acquisition Software for Single Particle Analysis (ThermoFisher)
Data analysis	Relion 3.0 was used for single particle processing, model building was performed using Phenix and Coot, Image processing was performed using UCSF-chimera<br><br>XRF analysis was performed using the raw capillary electrophoresis (CE) data using the QuShape Software package in combination with the BoXFP wrapper module

For manuscripts utilizing custom algorithms or software that are central to the research but not yet described in published literature, software must be made available to editors and reviewers. We strongly encourage code deposition in a community repository (e.g. GitHub). See the Nature Portfolio guidelines for submitting code & software for further information.

Data

Policy information about availability of data

All manuscripts must include a data availability statement. This statement should provide the following information, where applicable:

- Accession codes, unique identifiers, or web links for publicly available datasets
- A description of any restrictions on data availability
- For clinical datasets or third party data, please ensure that the statement adheres to our policy

The data that supports the findings of this study are available from the corresponding authors upon request. Correspondence and requests for materials should be addressed to N.P or P.G.S. The data underlying graphs are provided as supplemental datasets. The atomic coordinates for HBV NC were deposited in the Protein

Data Bank with code 7ABL. The icosahedrally averaged, symmetry-expanded and focused classified on genome and asymmetric cryo-EM density maps were deposited in the EM Data Bank with codes EMD-11700, EMD-11702 and EMD-11701, respectively.

Field-specific reporting

Please select the one below that is the best fit for your research. If you are not sure, read the appropriate sections before making your selection.

☒ Life sciences ☐ Behavioural & social sciences ☐ Ecological, evolutionary & environmental sciences

For a reference copy of the document with all sections, see nature.com/documents/nr-reporting-summary-flat.pdf

Life sciences study design

All studies must disclose on these points even when the disclosure is negative.

Sample size	Triplicate repeats were performed for assemblies and X-Ray footprinting.
Data exclusions	No data were excluded
Replication	Data were collected on different samples ensuring reproducibility, with the exception of cryoEM reconstruction and X-Ray footprinting
Randomization	Samples could not be randomised as we are looking at specific structures and sequences affecting HBV assembly
Blinding	Samples are blinded using generic numerical labeling system to avoid bias

Reporting for specific materials, systems and methods

We require information from authors about some types of materials, experimental systems and methods used in many studies. Here, indicate whether each material, system or method listed is relevant to your study. If you are not sure if a list item applies to your research, read the appropriate section before selecting a response.

Materials & experimental systems

n/a	Involved in the study	
☒	☐	Antibodies
☒	☐	Eukaryotic cell lines
☒	☐	Palaeontology and archaeology
☒	☐	Animals and other organisms
☒	☐	Human research participants
☒	☐	Clinical data
☒	☐	Dual use research of concern

Methods

n/a	Involved in the study	
☒	☐	ChIP-seq
☒	☐	Flow cytometry
☒	☐	MRI-based neuroimaging
