# Supplementary material for: In vitro functional analysis of gRNA sites regulating assembly of hepatitis B virus
Source: Commun Biol. 2021 Dec 16;4:1407. doi: 10.1038/s42003-021-02897-2 (PMC8677749; doi:10.1038/s42003-021-02897-2)
Supplement: Supplementary file 10 — Description of Additional Supplementary Files [file 42003_2021_2897_MOESM10_ESM.pdf]

Description of Additional Supplementary Files

File name: Supplementary Data 1
Description: Light scattering data for NCP reassemblies around gRNA and ΔPS/PS1.

File name: Supplementary Data 2
Description: Normalised reactivity and ΔReactivity values plotted in Fig 5b & d.

File name: Supplementary Data S1
Description: Light scattering data and autocorrelation curve of NCPs expressed in *E. coli*.

File name: Supplementary Data S2
Description: Data for autocorrelation curves of gRNA, Δε, ΔPS and ΔPS1; and light scattering as a result of NCP reassemblies around Δε.

File name: Supplementary Data S3
Description: Light scattering data for NCP reassemblies around ΔPS2/3.
